# PTSD and Audio Vestibular Symptoms: A Two-Way Street Driven by the Amygdala? A Speculative Hypothesis

**DOI:** 10.3390/brainsci16030282

**Published:** 2026-02-28

**Authors:** Dalila Roccamatisi, Iole Indovina, Pietro De Luca, Massimo Ralli, Diego Kaski, Arianna Di Stadio

**Affiliations:** 1 Laboratory of Neuromotor Physiology, IRCCS Santa Lucia Hospital of Rome, 00179 Rome, Italy; dalilaroccamatisi@gmail.com (D.R.); i.indovina@hsantalucia.it (I.I.); dr.dlp@hotmail.it (P.D.L.); 2 Department of Systems Medicine, Centre for Space BioMedicine, University of Rome Tor Vergata, 00133 Rome, Italy; 3 Department of Medicine, UniCamillus International Medical University, 00131 Rome, Italy; massimo.ralli@unicamillus.org; 4 SENSE Research Lab, UCL Queen Square Institute of Neurology, London WC1N 3BG, UK; d.kaski@ucl.ac.uk; 5 Life Sciences, Health and Health Professions Department, LINK University, 00165 Rome, Italy

**Keywords:** post-traumatic stress disorders, PTSD, tinnitus, dizziness, vertigo, amygdala, neuroinflammation

## Abstract

**Background/Objective**: Auditory and vestibular (AV) symptoms can be considered functional neurological disorders (FND) when they do not arise from structural abnormalities. These symptoms can arise as expressions of underlying neuropsychological or psychiatric conditions, yet they may also play a role in precipitating or maintaining such disorders. This systematic review aimed at exploring the prevalence of AV symptoms in post-traumatic stress disorders (PTSD) as well as to understand if they correlate with each other. **Methods**: We conducted a systematic review of the literature including PubMed, Scopus and Google Scholar. Articles in English published between 1985 and 2025 were screened using the following keywords: “Tinnitus”, “Ghost sound”, “Dizziness” “Vertigo”, “Persistent Postural Perceptual Dizziness”, “PPPD”, “Hearing concerns”, “Hyperacusis”, “Diplacusis”, “Auditory hallucination(s)”, “Audio and Vestibular symptom” and “Post Traumatic Stress Disorders” or “PTSD”. Risk of bias was used to evaluate the quality of the articles. **Results**: We found 18 studies analysing specifically these symptoms in PTSD, including a total of 598,654 subjects. We identified 123.006 patients with PTSD (20.5% of the entire analysed sample) suffering from AV symptoms, in particular tinnitus (33.8%) and a combination of hearing loss and tinnitus (29%). A very small separate percentage suffered from auditory hallucinations (0.3%) or vertigo/dizziness (0.8%). Magnetic resonance imaging studies have identified hyperactivation in various brain areas both in PTSD and in the presence of AV symptoms, with amygdala hyperactivation emerging as the most common finding. **Conclusions**: Based on the results of this systematic review, patients with PTSD suffer from AV symptoms in 20.5% of cases. MRI studies conducted separately on patients with PTSD and tinnitus, vertigo or dizziness showed a hyperactivation of the amygdala in all these conditions. We speculate that amygdala hyperactivation might explain the coexistence of and the relationship between PTSD and AV symptoms.

## 1. Introduction

Auditory and vestibular (AV) symptoms have been observed in different psychiatric conditions, like schizophrenia and anxiety [1,2,3,4]. The presence and the origin of such symptoms have been linked to the altered perception of sensory stimuli [2,4]. To discern whether hearing or vestibular symptoms arise independently and then impact negatively on anxious states [5,6] or whether they are themselves symptoms of anxiety [2,4] can be challenging.

The hyperactivation of central auditory or vestibular pathways may result in an altered perception of sound, body orientation and balance, with a negative impact on quality of life if left untreated [2,5]. The presence of AV symptoms may exacerbate pre-existing psychological or psychiatric comorbidities. Compensatory mechanisms that should usually aid the resolution of symptoms can be influenced by psychological/psychiatric disorders and hinder recovery from audio vestibular disorders [7].

AV symptoms are typically classified as functional neurological disorders (FND) when they occur without an identifiable structural otological, neurological, or vestibular cause [8,9]. The presence of AV symptoms has been identified and confirmed in schizophrenia [2] and other psychiatric disorders [3,4]; however, how these symptoms relate to post-traumatic stress disorders (PTSD) remains underexplored.

PTSD induces hyperactivation across diffuse brain areas, leading to hyperarousal states [10] that could induce AV symptoms. However, to date, no studies have specifically evaluated the presence or incidence of AV in PTSD.

We speculate that AV symptoms might increase the “fear” sensation that worsens PTSD, creating a cycle that may be difficult to break, and that, in this population, AV symptoms’ incidence might be underestimated.

In the context of fear conditioning, reduced habituation to aversive stimuli has also been observed in persistent postural perceptual dizziness (PPPD) [11], an FND that may develop following an acute vestibular episode and persist after the initial event has resolved [12], in a manner analogous to PTSD.

This systematic review of the literature aims to evaluate (a) the presence and type of AV symptoms and their prevalence in a large PTSD population, (b) their relationship with PTSD and (c) possible treatment options.

## 2. Materials and Methods

**Figure 1:**
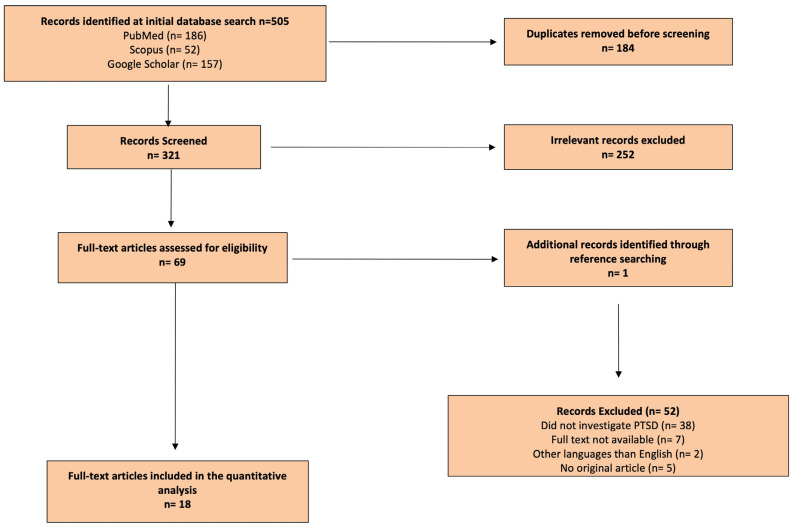
Literature search process (Preferred Reporting Items for Systematic Reviews and Meta-Analyses flow diagram).

We performed a systematic review of the literature in accordance with the Preferred Reporting Items for Systematic Reviews and Meta-analysis (PRISMA) checklist (Supplementary Material File S1) and statement recommendations (Figure 1). The review of the literature was performed between June and December 2025. We analyzed scientific literature published between 1985 and 2025 available on PubMed, Scopus and Google Scholar. This review did not include a meta-analysis of the collected data. Due to the nature of this review, Institutional Review Board approval was not necessary.

### 2.1. Search Strategy

A comprehensive search strategy, developed in partnership with a medical librarian, was performed on PubMed, Scopus and Google Scholar without time restrictions. The keywords used were: “Tinnitus”, “Ghost sound”, “Dizziness”, “Vertigo”, “Persistent Postural Perceptual Dizziness”, “PPPD”, “Hearing concerns”, “Hyperacusis”, “Diplacusis”, “Auditory hallucinations(s)”, “Audio and Vestibular symptom” and “Post Traumatic Stress Disorders” or “PTSD”. Only articles in the English language were considered for the analysis.

Two independent investigators (DR and PDL) reviewed the articles extracted from the literature review. Duplicates were removed, and then each reviewer individually filled in an Excel data sheet (Microsoft Corporation, Redmond, WA, USA) including information extracted from the articles. Files were then compared, and disagreements on the inclusion/exclusion papers were discussed until complete agreement was reached by both researchers. Only papers that received full consensus were considered.

PRISMA guidelines were followed to conduct the systematic review, and the full list of references was screened for potentially relevant articles. Whilst following PRISMA guidelines, this review was not formally registered on PRISMA.

### 2.2. Study Selection Criteria

We included articles with the following characteristics: patients (20–75 years) suffering from tinnitus alone or combined with vertigo or dizziness and PTSD written in the English language, with the full text available. Other conditions such as depression or psychosis were excluded. Selected articles were read in full to assess the study objectives and the level of evidence.

### 2.3. Data Extraction

A spreadsheet was completed using the data extracted from the articles read in full by the researchers. The following information was included: name of the author, year of publication, type of study, country where the study was conducted, number of subjects analyzed, patients’ characteristics, auditory results, treatment, outcome, presence or absence of the comparison group, characteristics of control groups.

### 2.4. Risk of Bias Assessment

The National Institutes of Health’s (NIH) quality assessment tools for Observational Cohort and Cross-Sectional Studies and for Case-Control studies were used to assess the risk-of-bias checklists, due to the different study designs (https://www.nhlbi.nih.gov/health-topics/study-quality-assessment-tools, accessed on 30 December 2025). The rating of each study was categorized as poor, fair or good (i.e., unbiased and fully described). Two authors (A.D.S., P.D.L.) independently gave a score to each article, and any disagreement was resolved following focused discussion between the researchers. The main difficulty in judging the articles was identified for the “fair” studies; in this case, if one of the authors judged the article as “good”/“poor” and the other as “fair”, a third author (I.I.) was invited for an additional evaluation. Based on the judgement of the third author, the article was scored as “fair”, “good” or “poor” based on the agreement of 2/3 evaluators.

## 3. Results

### 3.1. Details of Included Studies

PRISMA plot (Figure 1) shows the results obtained by applying keywords and study selection criteria.

Eighteen articles were identified and included in this systematic review. Of these articles, 12 (66.6%) were prospective studies, of which one was a case-control. Five (27.7%) studies were retrospective, one of which was a case-control, and one (5.7%) a longitudinal study. Of the eighteen studies, twelve (66.6%) were performed in the United States of America (USA), two (11.1%) in the United Kingdom (UK), one (5.5%) in Germany, one (5.5%) in Denmark, one (5.5%) in Australia, and one in Turkey (5.5%) (Table 1).

Five studies reported auditory hallucinations, four tinnitus, two hearing loss (HL) and tinnitus, five dizziness, and two studies dizziness/vertigo and HL.

Ten articles (55.5%) were classified as good quality, 5 (27.7%) as fair and 3 (16.8%) as poor (Table 2).

### 3.2. Details About Population Included in This Study

Eleven (61.1%) studies included veterans as the study population, two psychiatric population (11.1%), one (5.5%) veterans and military, one (5.5%) military, one (5.5%) refugee, one (5.5%) interpersonal assault (family, couple violence), one (5.5%) earthquake.

A total of 598,654 subjects were included. Most subjects participated in three studies [21,23,26] that did not specify the gender (570,492 individuals); of the remaining 28,162, 8078 (28.7%) were women. Based on available data from studies in which age was reported, only two studies [17,21] described a population over 50 years old.

**Figure 2:**
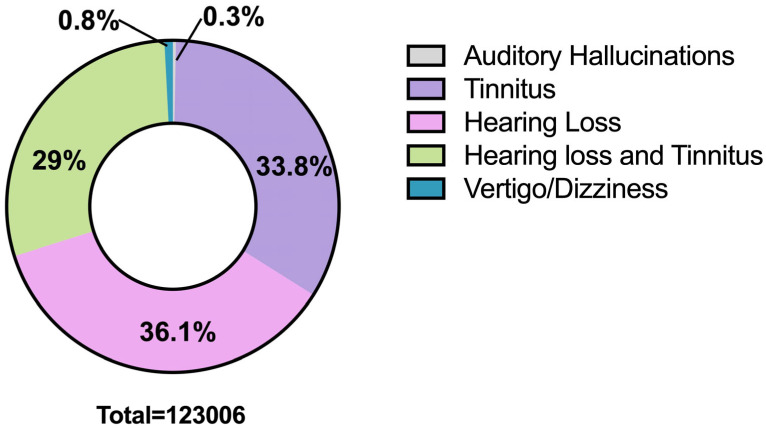
Symptom distribution in subjects that manifested AV symptoms with PTSD. Percentages relate to the sample of patients who presented AV symptoms.

A total of 123,006 individuals (20.5% of the entire sample) described AV symptoms (Figure 2). Of these, 44,510 subjects were exclusively affected by HL, 41,424 had tinnitus only, 35,702 suffered from HL and tinnitus, 1010 had dizziness/vertigo exclusively, and 360 reported auditory hallucinations (Table 3). There was no specific information about sex distribution, comorbidities or increased risk.

The auditory hallucinations consisted of pure sounds, not of verbal sounds. The studies that discussed vertigo/dizziness did not refer to Persistent Postural-Perceptual Dizziness (PPPD). We did not identify any study on PPPD and PTSD specifically.

## 4. Discussion

### 4.1. Analysis of Included Study

We identified a total of 18 articles that discussed the presence of audio vestibular symptoms related to a population of patients with PTSD. Perhaps unsurprisingly, the identified articles had a range of different study designs, including prospective, retrospective and case series. Only two studies, one prospective and one retrospective study, evaluated the presence of the AV symptoms comparing people with PTSD with a healthy population. The remaining 16 studies only reported the presence of AV in people with PTSD without comparative analyses. As such, it was only possible to report the presence of AV symptoms and carry out more descriptive analyses.

Seven studies reported the presence of vestibular disorders, described as vertigo or dizziness, six reported data on tinnitus, five referred to auditory hallucinations, and four contained information about HL. It was not possible to perform statistical analyses to probe the prevalence of a specific symptom.

A main outcome of this systematic review is the need for a well-structured comparative study of a PTSD population versus a healthy population to correctly identify the real prevalence of AV symptoms in PTSD, to specify the predominant symptom type and to evaluate the risk of experiencing AV symptoms compared to a healthy population.

### 4.2. Analysis of Population

Overall, analysis of the data extracted from this systematic review showed that 20.5% of individuals with PTSD experienced auditory hallucinations, hearing loss, tinnitus, dizziness and vertigo, either alone or in various combinations.

HL was the most common symptom (36.2%), followed by tinnitus (33.8%) and a combination of HL and tinnitus (29%). Dizziness and auditory hallucinations were the lower represented symptoms in our study, accounting for 0.8% and 0.3% of the total, respectively.

The consistent incidence (36.2%) of HL was expected, given the characteristics of the population studied. However, the possible involvement of central HL after head concussion [31], which is common among veterans and war-exposed individuals, could not be evaluated because the studies provided only basic audiological data. Consequently, HL was considered solely of peripheral origin and was not discussed further, since it lies outside the scope of this review.

The percentage of tinnitus (33.8%) did not include the studies where tinnitus was generically analysed as “auditory hallucination” because in these types of studies the occurrence of tinnitus was not quantifiable. For this reason, the percentage occurrence of tinnitus described in this work could be underestimated. Notably, the presence of AV symptoms increased with the increase in frequency of past traumas, particularly for auditory hallucinations and tinnitus [29]. Regarding tinnitus in individuals with HL (29%), it is important to note that a peripheral cause (damage to cochlear hair cells) is responsible for the ghost sound only during the first weeks after the acoustic trauma. Beyond 4–6 months, a persistence of the sound is attributed to the hyperactivation of the auditory cortex [32]. For this reason, tinnitus in veterans may be considered functional, even in the presence of hearing loss [29].

Dizziness/vertigo had a very low incidence in our study; however, we believe that this data may be underestimated and that the low incidence (0.8%) was in fact related to the small size of the sample (1010). In fact, Radziej et al. showed that both organic and functional vestibular symptoms worsen in the presence of PTSD [33]. Studies on larger samples must be conducted to correctly understand the incidence of vertigo/dizziness in PTSD.

Auditory hallucinations were the lowest represented symptom (0.3%). However, even in this case, a very small sample was analysed for this symptom, and we suspect that this could have impacted on its real incidence. It must be also considered that tinnitus itself could be a “phantom sound” and have been included among auditory hallucinations. We recommend that large, systematic studies dividing verbal and sound hallucinations could better clarify the prevalence of tinnitus or auditory hallucination in PTSD.

### 4.3. MRI Studies in PTSD, Tinnitus and Vertigo and Neuroinflammation

MRI studies in PTSD found smaller hippocampal and anterior cingulate (ACC) volumes but inconsistent hyper- or hypoactivity in the medial prefrontal cortex, along with a hyperactivation of the amygdala and superior temporal gyrus (STG) [34,35]. The orbitofrontal cortex is generally hypoactive [34] but shows hyperactivity in PTSD with dissociation [35]. Notably, the amygdala, prefrontal cortex, hippocampus and hypothalamus are common targets of deep brain stimulation in the treatment of PTSD [34].

Orbitofrontal cortex hypofunction diminishes inhibitory control over the amygdala, thereby exacerbating fear processing [34,35]. Amygdala, STG and dorsal ACC (dACC) hyperactivity [34,35], with the STG regulating sound perception and the dACC assessing the salience of sensory stimuli [36], represent a combination of alterations that lead to a heightened perception of environmental stimuli and a heightened evaluation of the salience of non-harmful stimuli in PTSD [34].

Animal models have been used to investigate the neural correlates of tinnitus and hyperacusis, which demonstrate hyperactivity within both emotional and auditory networks [37]. Specifically, an fMRI study in rats, in which tinnitus and hyperacusis were induced via an ototoxic agent, revealed a hyperactivation of the auditory network (inferior colliculus, medial geniculate and auditory cortex) as well as the cerebellum, amygdala and reticular formation [37]. In human studies of patients with tinnitus, the brain’s ability to downregulate sound perception was found to be impaired. This deficit is linked to a hyperactivation of the right middle temporal gyrus, right superior frontal gyrus and right angular gyrus, alongside a hypoactivation of the left cuneus, right middle occipital gyrus and thalamus [38,39]. Furthermore, in individuals with chronic tinnitus, whether or not they have depression, decreased amygdala functional connectivity (FC) with the prefrontal cortex and anterior cingulate cortex has been observed, along with increased amygdala FC with the postcentral and lingual gyri in patients with depression relative to those without [40].

In summary, both tinnitus/hyperacusis and PTSD induce widespread hyperactivity in the auditory network, alongside diminished prefrontal regulation [34,35,38,39,40].

Analogies can be made between PTSD and PPPD, an FND in which chronic dizziness might appear in some cases because of an acute vestibular event but persist after the event has resolved [12]. Indeed, attempts have been made to relate PPPD to fear conditioning by studying the blink reflex to nociceptive stimuli, and lack of habituation in response to pain was found [11]. Although the symptoms of PPPD and PTSD differ, as patients with PTSD usually experience acute and severe episodes of vertigo or dizziness [19,22], whereas in PPPD dizziness is typically chronic and less intense [12], they may partially overlap depending on their intensity. In this review we did not identify studies directly linking PTSD to PPPD, perhaps hinting at a distinct pathophysiology across the two disorders. Although a hyperactivity of the vestibular network was initially expected in PPPD, the deactivation of vestibular areas has instead been observed [41]. However, a recent study showed that PPPD patients had stronger fMRI activation in response to galvanic stimulation in areas related to the vestibular network, such as the supramarginal gyrus, the parietal operculum (OP3) and the vermis, compared to a healthy control group [42]. In general, patients with PPPD exhibit hypervigilant postural control and increased visual dependence, supporting the hypothesis that PPPD arises from altered interactions among visuo-vestibular, sensorimotor and emotional networks. These changes lead to an overreliance on visual rather than vestibular inputs and enhance the influence of anxiety-related mechanisms on locomotor control and spatial orientation [41,43]. The comparison between healthy individuals, individuals with PPPD and those with anxiety disorders showed that PPPD have increased neural responses compared to the healthy controls in the anxiety network including the amygdala, insula, lentiform nucleus in the basal ganglia, hippocampus, inferior frontal gyrus (IFG) and brainstem [44].

The hyperactivation of the amygdala, STG and dACC in PTSD was associated with hyperperfusion in these areas [34,35]; chronic hyperperfusion, even at a subclinical level, may lead over time to endothelial dysfunction with an increased release of reactive oxygen species [ROS] [45]. Excessive production of ROS is a well-known trigger of neuroinflammation [46], which, if left untreated, may initiate a self-perpetuating cycle. In brain neuroinflammation dysfunctional (pro-inflammatory) microglia produce ROS which in turn further fuel the neuroinflammatory process [46]. Excessive production of ROS is also implicated in the neuroinflammation observed in several psychiatric and psychological disorders, whereas its role in PTSD has so far been only hypothesized [9,46,47,48]; some authors have proposed the use of anti-neuroinflammatory treatments to manage psychiatric disorders, based on the concept that increased ROS production is a key driver of neuroinflammation [49].

Recent studies investigating the immune system in PTSD identified an overactive immune response in these patients [47,50] with an increase of circulating pro-inflammatory elements [51] that can bypass the brain–blood barrier (BBB) [52], worsening symptoms and the progression of the condition [53]. Neuroinflammation, through a self-perpetuating mechanism, leads to the production of pro-inflammatory mediators that promote the activation of M1 (pro-inflammatory) microglia. M1 microglia, also referred to as disease-associated microglia (DAM), contribute to demyelination and neurodegeneration [54] Moreover, they release pro-inflammatory mediators that further amplify inflammation and promote the generation of additional harmful microglial cells.

fMRI studies, conducted separately in individuals with PTSD and in patients experiencing AV symptoms [39,40,41,42,44], have revealed hyperactivation in overlapping brain regions. Although this does not imply a direct correlation or association between PTSD and AV symptoms, it raises the possibility of shared underlying mechanisms.

A better understanding of this coexistence could be useful to identify treatments able to manage symptoms, many of which do not have effective remedies at present. It is worth considering whether the presence of AV symptoms in patients with PTSD may reflect active neuroinflammatory processes that could negatively impact PTSD outcomes.

### 4.4. The Role of Amygdala and Possible Therapeutical Options

The amygdala, a central structure in fear processing, modulates the salience of sensory information, including auditory and vestibular stimuli, via brainstem, thalamic and cortical inputs [55]. The amygdala receives projections from vestibular nuclei through the parabrachial nucleus in the brainstem [56,57]. The lateral nucleus of the amygdala communicates with other amygdaloid nuclei, particularly the basal and central nuclei, which in turn influence brainstem regions such as the locus coeruleus (LC) and cortical areas including the prefrontal cortex [55]. On the other hand, heightened sensory processing may lead to increased stress and anxiety levels [58]. The extent of this effect is influenced by individual life experiences and genetic susceptibility [58,59].

**Figure 3:**
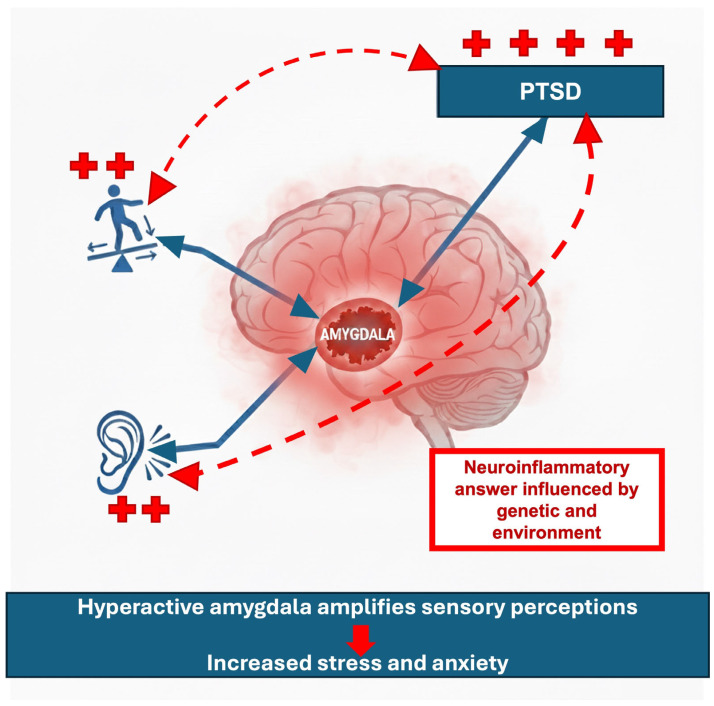
The image shows how the hyperactive amygdala might impact on the audio vestibular perception and the brain reaction to the stimuli causing the worsening of PTSD. The hyperactivity of the STG and dACC in PTSD may contribute to a self-reinforcing loop that amplifies auditory and vestibular processing. The solid blue lines represent links that are supported by empirical evidence and red dashed lines indicate hypothetical mechanisms. We hypothesize that dizziness (++) plus hearing symptoms (++) could negatively impact PTSD severity (++++).

The sensorium serves as the interface between the individual and the external environment, and disruptions in sensory processing can therefore impact everyday functioning. As the amygdala plays a central role in regulating attention toward salient sensory inputs, its hyperactivation in PTSD may alter sensory perception and contribute to auditory and vestibular symptoms commonly described in these patients. Alternatively, there may be a bidirectional association between PTSD and AV symptoms, governed by changes in the amygdala (Figure 3).

Some holistic and wellness-oriented treatments are extremely beneficial for people with PTSD, and these treatments can also influence sensory perception. For example, sunlight exposure reduces anxiety and improves patients’ symptoms [60]. Yoga, a traditional Eastern discipline that enhances self-perception and mindfulness, seems to be beneficial in PTSD [61]. Both treatments stimulate the senses, creating a positive experience and wellbeing. The latter produces endorphins that modulate and improve synaptic transmission [59]. The beta-endorphins [62] interact with microglia and glia function, reducing hyperexcitability and neuroinflammation [63]. As previously mentioned, reduced cellular activity leads to a lower production of ROS, which are inducers of M1 microglia [64], generally improving brain environment. This may represent one mechanism by which holistic treatments exert beneficial effects in patients with PTSD, although experimental evidence is required to validate it.

Along similar lines linking neuroinflammation and hyperactivation, it is reasonable to propose that medications that reduce synaptic transmission, thereby attenuating attentional engagement, may be beneficial in patients with PTSD as well as in those experiencing tinnitus and vestibular symptoms. These agents may reduce environmental sensory processing and dampen stimulus-driven activity within cortico-subcortical networks involving the amygdala. A less active amygdala [65] may, in turn, decrease overall brain activation and ROS production [51,52], which, based on the mechanisms outlined above, could contribute to a reduction of neuroinflammation.

Studies focusing on the neural structures and pathways involved in the processing of sensory stimuli in patients with PTSD should be performed to understand (i) if the use of multisensory positive stimuli might improve PTSD and (ii) the effects of combined hearing-aid rehabilitation and pharmacological treatment on PTSD symptoms in war veterans with hearing loss.

### 4.5. Study Limitations

This systematic review presents some limitations. Firstly, the included studies were heterogeneous; they presented differences in the characteristics of the observed sample (veterans, psychiatric, refugees), they were not stratified for gender, pre-existing conditions and/or risk factors, and they used different methods to collect and analyse the clinical data. Moreover, the studies had different designs (66.6% prospective, 27.7% retrospective) and had different sample sizes; the latter could have impacted the reported statistical analyses.

Regarding the sample selection, most of the studies analysed veterans. Former military service members have been exposed to blast sounds, head concussions and other trauma that could directly affect both hearing function and motor abilities, making it very difficult to understand the real origin of audio-vestibular symptoms, whether they are functional, macrostructural or a combination of both. Moreover, brain concussion is an important trigger of brain neuroinflammation [66]. Although neuroinflammation is a physiological process, in case of chronicity it becomes pathological. The chronicity of neuroinflammation, which is related to several individual factors [67], is responsible for a series of symptoms, including the onset of psychological and psychiatric disorders [53]. Another criticism is that auditory hallucinations have not been detailed in the studies, so a ghost sound like tinnitus could have been included among auditory hallucinations, rendering it difficult to well distinguish tinnitus from a more general ghost perception [26].

## 5. Conclusions

Our review showed that audio-vestibular symptoms can be observed in PTSD; tinnitus is the most common after HL. Dizziness/vertigo can be considered as the second more common symptoms in PTSD.

In several psychological/psychiatric conditions, AV symptoms are generally considered as an FND [7,8,68]; we identified their presence in a PTSD context. We speculate that sensory hyperactivity, which affects amygdala function in the case of both tinnitus and PTSD, might lead to neuroinflammation that can exacerbate PTSD establishing a self-perpetuating cycle.

Because tinnitus and dizziness/vertigo itself could be a source of stress and can exacerbate PTSD symptoms, thus worsening neuroinflammation, we suggest that the effective treatment of PTSD may require the rehabilitation of these altered sensory functions. The use of anti-inflammatory molecules associated with traditional drugs and ad hoc treatments such as vestibular rehabilitation and hearing desensitization might be a good option for a correct and complete taking in charge of patients with PTSD.

## Figures and Tables

**Table 1:** Details from each included study selected based on the keywords and the inclusion and exclusion criteria adopted for the systematic review.

Author, Year, Country	Design of the Study	Sample Size (*n*)	Gender (*n*)	Age (Mean, Year)	Cohort	Symptom/s	Findings
Mueser et al., 1987, United States [13]	Retrospective	36	36 M	Not available	Veterans	Auditory hallucinations	The preliminary observations suggest that the presence of auditory hallucinations in posttraumatic stress disorder relate to higher combat exposure and more severe PTSD. Moreover, veterans with auditory hallucination were more refractory to PTSD treatments.
Grigsby et al., 1993, United States [14]	Prospective	70	40 F, 30 M	34.7	Psychiatric population	Vertigo	10 patients presented vertigo associated with PTSD
Fagelson, 2007, United States [15]	Prospective	103	100 M, 3 W	Not available	Veterans	Tinnitus	Individuals with tinnitus and concurrent PTSD reported significantly poorer tinnitus self-efficacy and more handicapping tinnitus effects when compared to individuals with other psychological conditions or those with tinnitus alone. The sound-triggered exacerbation of tinnitus was more common for patients with PTSD than patients with tinnitus only.
Hoffer et al., 2010, United States [16]	Prospective	81 acute, 25 subacute, 42 chronic	81 (80 M, 1 W); 25 M; 42 unspecified	24, 26, 25	Military exposed to trauma	Dizziness, Vertigo, Hearing Loss (HL)	Among patients evaluated in the acute phase, dizziness was reported in 98% (*n* = 79), vertigo in 2% (*n* = 2), and HL in 33% (*n* = 27). In subacute and chronic conditions, 50 patients reported dizziness, 25 vertigo, and 14 HL. PTSD was observed in 2% of acute cases, 20% of subacute cases, and 44% of chronic patients.
Liu et al., 2015, United States [17]	Retrospective	94	93 M, 1 W	62	Veterans	Dizziness, Vertigo, Hearing Loss (HL)	90 had HL, 74 had tinnitus, 24 had vertigo, and 77 had PTSD. The result of Tinnitus Handicap Index (THI) showed that THI < 38 was associated with PTSD in 58.8% of patients, whether >38 with 91.8% PTSD.
Geddes et al., 2016, United Kingdom [18]	Prospective	106	27 W, 79 M	34.4	Interpersonal assaulted patients	Auditory hallucinations	Cognitive processing during trauma (lack of self-referential processing and dissociation), beliefs about permanent negative change, self-vulnerability and self-blame and cognitive response styles (thought suppression, rumination and numbing) were significant predictors of later hallucinations. The way in which trauma is processed may partly determine the occurrence of hallucinations.
Haber et al., 2016, Germany [19]	Prospective case-control	50	3 W, 47 M	43	Veterans	Vestibular symptoms	The findings indicate that veterans with worse PTSD symptoms report increased vestibular related symptoms. Additionally, veterans with PTSD reported three times more dizziness-related handicap than veterans without PTSD. Veterans with increased avoidance reported more vertigo and dizziness-related handicap than those with PTSD and reduced avoidance.
Nygaard et al., 2017, Denmark [20]	Retrospective case-control	181	77 W, 104 M	45	Refugees	Auditory hallucinations	Most symptoms identified were auditory hallucinations (66.2%; 120 people) and persecutory delusions (50.0%).
Swan et al., 2017, United States [21]	Retrospective	570,248	Not available	57.1	Veterans	Tinnitus +/− HL; HL +/− tinnitus	7.78% (44,379) of the patients were diagnosed with hearing loss alone, 6.54% (37,306) with tinnitus alone, and 6.24% (35,583) with both hearing loss and tinnitus. Comorbid TBI, PTSD, and depression were significantly associated with increased rates of hearing loss, tinnitus, or both conditions together. Older individuals, males and those with TBI, PTSD or vertigo/dizziness were significantly more likely to have hearing loss, tinnitus or both. To provide more holistic post-deployment support, this multitude of conditions should be carefully considered in the planning of clinical care and beyond.
Fox et al., 2019, United States [22]	Prospective	50	4 W, 46 M	49.4	Veterans	Dizziness	Ninety percent of participants scored above the Vertigo Symptom Scale (VSS) threshold, suggesting “severe dizziness.” The most endorsed symptom on the VSS was “headache or pressure in the head”.
Hinton, 2020, United States [23]	Prospective	90	not available	Not available	Veterans	Auditory hallucinations	The survey revealed that 42% (38/90) had auditory hallucinations (AHs) in the last month. Of those with AHs, 87% (33/38) had PTSD, whereas, of those without AHs, 31% (16/52) had PTSD, giving a chi square of 27.8, *p* < 0.001, odds ratio 14.8 (4.8–45). Most AHs were of a “ghost summoning” (khmaoch hao), considered an exhortation to go with a ghost (e.g., hearing “Please come with me, younger sister”), experienced by 73% percent of patients with AHs. The voices were always exterior and usually loud and clear. AHs were heard most often during hypnagogia (i.e., upon falling asleep or awakening), experienced by 72% of patients with AHs, whereas 28% of patients with AHs experienced AHs when fully awake.
MacGregor et al., 2020, United States [24]	Prospective	1026	51 W, 975 M	Not available	Veterans	Tinnitus	Those with battle blast injury had the highest prevalence of tinnitus, with 19.1% (196) and 31.3% (318 people) on the first and second health assessments, respectively. In a multivariate model adjusting for combat exposure, concussion, post-traumatic stress disorder and other covariates, tinnitus was associated with lower self-rated health for both the first (odds ratio [OR] = 3.31, 95% confidence interval [CI] = 2.07–5.30, *p* < 0.001) and the second assessment (OR = 2.52, 95% CI = 1.76–3.61, *p* < 0.001).
Terhaag et al., 2021, Australia [25]	Longitudinal	523	38 W, 343 M	47.4	Veterans	HL and tinnitus	More than half of veterans on PTSD treatment self-reported doctor-diagnosed hearing loss or tinnitus (277 subjects), whereas 43% (119) reported both. However, 75% reported subjective mild to moderate hearing impairment, and only 1% reported severe impairment. Service-related factors, such as longer length of service and exposure to explosions, were risk factors for having any hearing condition.
Moring et al., 2022, United States [26]	Retrospective	112	Not available	Not available	Veterans	Tinnitus	Half of participants with tinnitus demonstrated severe impairment. Correlational analyses indicated that reexperiencing, avoidance, negative emotions and cognitions and hyperarousal PTSD symptoms were significantly related to many factors of tinnitus-related distress, including intrusiveness of tinnitus, perceived loudness, awareness and annoyance. Participants with severe tinnitus demonstrated significantly greater reexperiencing, negative mood/cognitions, hyperarousal and PTSD total severity compared to those with mild or moderate tinnitus.
Sonstroem et al., 2023, United States [27]	Prospective	916 (424 Military and 492 Veterans)	Military: 271 M, 153 W; Veterans: 417 M, 75 W	34.6 Military: 34.1 Veterans	Military and Veterans	Dizziness	Overall, 22.4% of military (95 subjects) and 30.5% of veterans (150 people) self-reported dizziness. Compared to those with neither TBI nor blast exposure history, both service members and veterans with TBI (with or without blast) were three to four times more likely to self-report dizziness
Cengiz et al., 2024, Turkey [28]	Prospective	1004	680 W, 324 M	28.55	Earthquake	Dizziness	480 people presented dizziness, and dizziness was correlated with worse PTSD
Dudley et al., 2024, United Kingdom [29]	Prospective	67	34 W, 33 M	40.8	Psychiatric population	Auditory hallucinations	There were high levels of reported auditory (89.6%; 60 people), visual (58.2%) and tactile (46.3%) hallucinations. Hallucinations in two or more modalities were the norm (71.6% of participants). The number of hallucination modalities was moderately associated with a greater number of past traumas and PTSD symptoms. The linkage between trauma and auditory hallucinations extends to other sensory domains.
Geronimo-Hara et al., 2025, United States [30]	Prospective	23,830	6891 W, 16,639 M	Not available	Veterans	Tinnitus	New-onset tinnitus was self-reported by 10.7% (*n* = 2527) of participants, while 3.5% (*n* = 511) had a medical record tinnitus diagnosis. Tinnitus risk was associated with multiple characteristics, including active-duty service, being a member of the Army or Marine Corps, combat deployment experience, combat specialist occupation, prior history of mild traumatic brain injury, panic/anxiety, posttraumatic stress disorder (PTSD) alone, and PTSD comorbid with depression.

AHs: Auditory Hallucinations; CI: Confidence Interval; HL: Hearing Loss; OR: Odds Ratio; PTSD: Post Traumatic Stress Disorder; TBI: Traumatic Brain Injury; THI: Tinnitus Handicap Index.

**Table 2:** The quality of each included study based on Risk of Bias Assessment.

Author, Year, Country	Quality Score Assessment
Mueser et al., 1987 [13], United States	Poor
Grigsby et al., 1993 [14], United States	Fair
Hoffer et al., 2010 [16], United States	Good
Liu et al., 2015 [17], United States	Fair
Geddes et al., 2016 [18], United Kingdom	Good
Haber et al., 2016 [19], Germany	Good
Fagelson, 2017 [15], United States	Poor
Swan et al., 2017 [21], United States	Fair
Fox et al., 2019 [22], United States	Good
Nygaard et al., 2017 [20], Denmark	Good
Hinton, 2020 [23], United States	Fair
MacGregor et al., 2020 [24], United States	Good
Terhaag et al., 2021 [25], Australia	Good
Moring et al., 2022 [26], United States	Poor
Sonstroem et al., 2023 [27], United States	Good
Dudley et al., 2024 [29], United Kingdom	Fair
Geronimo-Hara et al., 2025 [30], United States	Good
Cengiz et al., 2024 [28], Turkey	Good

**Table 3:** Distribution of symptoms across studies included in this systematic review.

	Mueser et al. 1987 [13]	Grigsby et al. 1993 [14]	Fagelson et al. 2007 [15]	Hoffer et al. 2010 [16]	Liu et al. 2015 [17]	Geddes et al. 2016 [18]	Haber et al. 2016 [19]	Nygaard et al. 2017 [20]	Swan et al. 2017 [21]	Fox et al. 2019 [22]	Hinton et al. 2020 [23]	MacGregor et al. 2020 [24]	Terhaag et al. 2021 [25]	Moring et al. 2022 [26]	Sonstroem et al. 2023 [27]	Cengiz et al. 2024 [28]	Dudley et al. 2024 [29]	Geronimo et al. 2024 [30]	Total
Auditory Hallucinations	36	n/a	n/a	n/a	n/a	106	n/a	120	n/a	n/a	38	n/a	n/a	n/a	n/a	n/a	60	n/a	360
Tinnitus	n/a	n/a	103	n/a	74	n/a	n/a	n/a	37,306	n/a	n/a	514	277	112	n/a	n/a	n/a	3038	41,424
Hearing loss + Tinnitus	n/a	n/a	n/a	n/a	n/a	n/a	n/a	n/a	35,583	n/a	n/a	n/a	n/a	n/a	n/a	n/a	n/a	n/a	35,583
Hearing loss	n/a	n	n/a	41	90	n/a	n/a	n/a	44,379	n/a	n/a	n/a	n/a	n/a	n/a	n/a	n/a	n/a	44,510
Vertigo/ Dizziness	n/a	10	n/a	156	24	n/a	50	n/a	n/a	45	n/a	n/a	n/a	n/a	245	480	n/a	n/a	1010

n/a: indicates the symptom(s) was not analyzed in the context of the study.

## Data Availability

Not applicable due to the nature (systematic review) of the article.

## Supplementary Materials

The following supporting information can be downloaded at: https://www.mdpi.com/article/10.3390/brainsci16030282/s1, File S1: PRISMA 2020 checklist.

## Abbreviations

The following abbreviations are used in this manuscript:

PTSD	Post Traumatic Stress Disorder
AV	Audio Vestibular
PPPD	persistent postural perceptual dizziness
STG	Superior Temporal Gyrus
dACC	Dorso Anterior Cingulate Cortex
